# Alterations in Coronary Resistance Artery Network Geometry in Diabetes and the Role of Tenascin C

**DOI:** 10.31083/j.rcm2401006

**Published:** 2023-01-04

**Authors:** Attila Kiss, Gyorgy L Nadasy, Alexander Fees, Zsuzsanna Arnold, Ibrahim Aykac, Christopher Dostal, Gábor T Szabó, Petra Lujza Szabó, Maria Szekeres, Peter Pokreisz, Laszlo Hunyady, Bruno K Podesser

**Affiliations:** ^1^Ludwig Boltzmann Institute for Cardiovascular Research at the Center for Biomedical Research, Medical University of Vienna, 1090 Vienna, Austria; ^2^Department of Physiology, Faculty of Medicine, Semmelweis University, 1094 Budapest, Hungary; ^3^Kansas State University, Manhattan, KS 66506, USA; ^4^Department of Morphology and Physiology, Faculty of Health Sciences, Semmelweis University, 1088 Budapest, Hungary

**Keywords:** diabetes, microvascular dysfunction, resistance coronary artery network, Tenascin C, wall thickness

## Abstract

**Background:**

Geometrical alterations in the coronary resistance artery network and the potential involvement of Tenascin C (TNC) extracellular matrix protein were investigated in diabetic and control mice.

**Methods:**

Diabetes was induced by streptozotocin (STZ) injections (n = 7–11 animals in each group) in Tenascin C KO (TNC KO) mice and their Wild type (A/J) littermates. After 16–18 weeks the heart was removed and the whole subsurface network of the left coronary artery was prepared (down to branches of 40 μm outer diameter), *in situ* pressure-perfused and studied using video-microscopy. Outer and inner diameters, wall thicknesses and bifurcation angles were measured on whole network pictures reconstructed into collages at 1.7 μm pixel resolutions.

**Results:**

Diabetes induced abnormal morphological alterations including trifurcations, sharp bends of larger branches, and branches directed retrogradely (*p *< 0.001 by the χ^2^ test). Networks of TNC KO mice tended to form early divisions producing parallelly running larger branches (*p *< 0.001 by the χ^2^ probe). Networks of coronary resistance arteries were substantially more abundant in 100–180 μm components, appearing in 2–5 mm flow distance from orifice in diabetes. This was accompanied by thickening of the wall of larger arterioles (>220 μm) and thinning of the wall of smaller (100–140 μm) arterioles (*p *< 0.001). Blood flow should cover larger distances in diabetic networks, but interestingly STZ-induced diabetes did not generate further geometrical changes in TNC KO mice.

**Conclusions:**

Diabetes promotes hypertrophic and hypotrophic vascular remodeling and induces vasculogenesis at well defined, specific positions of the coronary vasculature. TNC plays a pivotal role in the formation of coronary network geometry, and TNC deletion causes parallel fragmentation preventing diabetes-induced abnormal vascular morphologies.

## 1. Introduction

Microvascular damage is one of the major severe consequences of diabetes. Diabetic microvascular pathology is characterized by uneven lumen diameter and increased wall thickness [1], local narrowing and dilation with microaneurysms prone to rupture [2, 3, 4], and tortuosity [2, 3, 5]. These conditions certainly hamper local hemodynamics. Histologically, loss of smooth muscle cells, accumulation of collagen and other connective tissue elements, basement membrane thickening, endothelial damage with impaired endothelial dependent dilation and increased permeability are characteristic of diabetes [6, 7]. Statistical-geometric analysis of the retinal microvasculature can be diagnostically important in diabetes [8], however, direct observation of coronary resistance artery geometry in the left ventricular tissue remains uninvestigated. Evidence suggests that a substantial part of the diabetic cardiomyopathy may be attributable to pathological alterations of the coronary resistance arteries. Clinically, reduced coronary flow reserve likely involves diabetic damage to the microvasculature [9, 10, 11]. Thickening of the basement membrane, thickening of the arteriolar media, perivascular fibrosis and microaneurysms [12, 13, 14] have been shown in coronary resistance arteries in diabetic patients and in animal models of diabetes. It is clinically well known that the angiographic macroscopic picture of such coronary angiograms mimics a “winter tree”, with no leaflets on the tree but only the large conductance vessels left. This is paralleled by a pronounced microangiopathy leading to the typical picture of diabetes-induced diffuse fibrosis and cardiomyopathy [12, 13, 14].

With pressure arteriography, Lynch *et al*. [15] did not find wall thickening in the 50–150 μm diameter range of coronary arterioles of diabetic patients. While evidence of microvascular wall damage and impaired endothelial function [16] are accumulating, less is known about potential alterations in coronary resistance artery network geometry in diabetes. Microaneurysms, spasms, and spiral deformations have been found via plastic filling in diabetic patients [12]. The pathological significance of resistance artery geometry alterations has been demonstrated previously by a significantly elevated number of geometrical disturbances with both age [17] and angiotensin II-induced hypertension in rats [18]. A sex-dependent remodeling of coronary resistance artery geometry was found in male and female rats after 12 weeks of heavy physical exercise [19]. However, the exact mechanism of adverse remodeling of small coronary arteries geometry due to diabetes are not fully know, there are substantial mechanisms are considered to play a role in. For example, there is eveidence that the impaired glucose metabolism and insulin resistance may contribute to substantial increase of resistance arterial wall thickness, fibrosis and vascular remodeling [20]. In addition, diabetes increases the risk of heart failure (HF), independent of coronary artery disease and other comorbidities. This pathological outcome, termed “diabetic cardiomyopathy”, is characterized by initial impairment of left ventricular (LV) diastolic function, structures and endothelial dysfunction. Indeed, these alterations may also facilite the geometrical changes of coronary resistance artery netowork in diabetes. The hallmark of extracellular matrix remodeling (ECM) and subsequently the increase of cardiac and perivascular fibrosis are often observed in diabetes. More recently, our group have investigated the pathophysiological role of Tenascin C, an ECM glycoprotein in (TNC) in macrovascular complications, e.g., the progression of aortic aneurysm [21] and pulmonary artery hypertension [22]. In general, TNC plays a role in development of various organs and tissue, but TNC re-expressed is high levels in tumor tissue as well as in chronic inflammation foci accompanied by fibrosis [23, 24, 25]. Furthermore, it has been identified as an essential component and mediator of adverse cardiac remodeling [26, 27, 28], hypertrophy and heart failure [29]. Accordingly, TNC KO mice show a significant less amount of fibrosis and impaired cardiac function [29], vascular remodeling [21]. TNC re-expression can also be localized to intimal hyperplasia, pulmonary artery hypertension, abdominal aortic aneurysm, renal dysfunction, renal transplant vasculopathy, and varicose veins and has been linked to worse clinical outcome [30, 31]. More important, recent clinical studies also demonstrated that high serum TNC levels were associated with worse cardiovascular outcomes and higher risk for acute coronary syndrome in diabetic patients [32, 33]. However, it is still unknown whether the alterations of TNC expression in diabetes is a bystander or plays a causative role in diabetes associated organs damage, cardiac and vascular dysfunction.

The aim of the current study was to characterize whether streptozotocin-induced diabetes in mice yields significant geometrical remodeling of the coronary resistance artery system and the effects of TNC on the coronary resistance artery network.

## 2. Materials and Methods

### 2.1 Animals

Adult (8–10 weeks old) male TNC KO mice (KO, RBRC00007 A, Experimental Animal Division, Tsukuba, Japan) and their wild-type littermates (Wt, A/J, #000646, The Jackson Laboratory, Sacramento, CA, USA) were used [34, 35, 36]. All animals received a standard laboratory care and were housed in air-conditioned rooms at 22 °C with a 12/12 h day/night cycle, including free access to water and standard mouse chow. The experimental protocol was approved by the regional Ethics Committee for Laboratory Animal Experiment conforming with the Guide for the Care and Use of Laboratory Animals published by the US National Institutes of Health (NIH Publication No. 85–23, revised 1996).

Streptozotocin (STZ; 50 mg/kg) was injected intraperitoneally into model group mice for five consecutive days. All mice were weighted accurately prior to STZ injection. STZ was weighted according to the body weight and dissolved in sterile Dulbecco’s PBS (DPBS, Gibco, Life Technologies Ltd, Basel, Switzerland). Because STZ should be degraded within 20–30 min, the STZ solution was prepared immediately before use then injected within 5 min. The STZ solution was freshly prepared on daily base. On experimental day 6, blood glucose was monitored via tail vein blood withdrawal as described previously [37]. Mice were considered diabetic if blood glucose levels show >15 mmol/L. Age-matched mice injected with sterile DPBS served as non-diabetic controls. Mice were given unlimited food and water and were not supplemented with insulin or anti-hyperglycaemic agents. Mice were sacrifed 16–18 weeks after the induction of diabetes. During the observation period (16–18 weeks) one A/J and two TNC KO diabetic animals were found dead Finally, Wt non-diabetic (n = 11), Wt diabetic (n = 9), TNC KO non-diabetic (n = 10) and TNC KO (n = 7) diabetic mice were used for further analysis.

### 2.2 In Situ Coronary Resistance Artery Network Geometry

After 16–18 weeks, blood pressure was measured in anesthetized animals (45 mg/kg pentobarbital, i.p.) in the right carotid artery, then the animals were exsanguinated, the whole vascular system was perfused with heparinized Krebs-Ringer solution and the heart was removed. The vascular network of the left coronary artery which in mice is running under the ventricular surface was carefully microprepared for easy visibility, left *in situ*, and the orifice was cannulated. The whole network was perfused with warm, oxygenated Krebs-Ringer solution at pressures of 70–100 mmHg using a servo-controlled pump (Living Systems, Burlington, VT, USA). Networks not able to keep at that pressure were discarded. Vessels down to 40 μm outer diameter were visualized. With proper adjustment of the illuminating light no staining should be applied. The advantage of the technique is that living pressurized vessels can be studied with oxygenized, temperature and pH controlled physiological salt solution flowing in the lumen. A substantial limitation is however, that diameter changes induced by pulsatile pressures in the aorta and contracting ventricular muscle will not be measured. Video-microscopic pictures of the pressurized network with saline flow in their lumen were made at small and large magnifications perpendicularly to the surface. A horizontally extended collage of the whole network was constructed from large magnification pictures at a resolution of 1.7 μm/pixel. This resolution was sufficient to spot biologically significant alterations in diameter and wall thickness even in the arteriolar range. Higher measuring accuracy can be reached on histological sections, but such are not pressurized, and are deformed by the fixation process. Higher accuracy is not needed as individual cells and collagenous bands have a few micrometer diameter, measured values can be statistically evaluated, anyway. Segments and bifurcations were numbered, and inner and outer diameters, wall thicknesses, and bifurcation angles were measured. The whole network was theoretically divided into 50 μm ring units, and their distribution was analyzed by methods as described earlier for rat coronary resistance artery networks [17, 18, 19, 38]. Briefly, each network was supposed to be composed of such 50 μm long cylindrical ring units, each of them characterized by the following data, outer diameter, inner diameter, wall thickness, direction of axis, location (of midpoint) in a coordinate system determined by the orifice and apex, direct distance from the orifice and distance from the orifice following the route of blood flow. As a sum 9646 ring units were studied in the four groups. In addition, all bifurcations were identified, daughter branches measured, and diameters were checked for adherence to the Murray-law. The Murray–law states that cube of the lumen diameter of the mother branch ($D_{\text{m}}$^3^) should be equal with the sum of the cubes of lumen diameters of the daughter branches ($D_{\text{d1}}$^3^+$D_{\text{d2}}$^3^).

### 2.3 Heart Dimensions

Transversal histological sections at the middle of the orifice-apex distance were prepared after formaldehyde (Formaldehyde solution 4%, Merck, Darmstadt, Germany) fixation and conventional hematoxylin-eosin staining was performed for left ventricular geometry assessment.

### 2.4 Statistics

Values are expressed as means ± SEM. One and two-way ANOVA was used for comparisons followed by Tukey post hoc analysis. Number of elements in different categories were compared with the χ^2^ probe. Scatters were compared with the F probe. Uniformly, *p *≤ 0.05 was accepted as limit for significance.

## 3. Results

### 3.1 Animal Characteristic 

Body weight and heart geometry are shown in Table 1. Body weight decreased after STZ the injection in Wt and TNC KO mice compared to controls, respectively (*p *< 0.001 and *p *< 0.05, two-way ANOVA). Blood pressure and blood glucose levels (STZ-induces diabetic groups; data not shown) were not different between the groups. Transversal diameter of diabetic hearts was significantly less in the TNC KO group, while diabetes decreased the thickness of the hind ventricular wall in both genetic groups and thickness of the septum in the Wt animals (Table 1).

**Table 1.** **Physiology parameters and heart geometry**.

	A/J		TNC KO		Two-way ANOVA
	Non-diabetic	Diabetic	Non-diabetic	Diabetic	
Body weight (gramm)	22.69 ± 0.97	20.52 ± 0.82	21.96 ± 0.51	17.25 ± 0.73	^###^, ^†^
Arterial blood pressure (mmHg)	80.5 ± 5.0	67.1 ± 4.6	77.9 ± 4.2	73.2 ± 2.5	n.s.
Heart					
Orifice-apex long axis (mm)	5.93 ± 0.23	5.75 ± 0.24	5.84 ± 0.29	5.25 ± 0.22	n.s.
Transversal (mm)	4.86 ± 0.12	4.42 ± 0.08	4.91 ± 0.19	4.19 ± ${\mathrm{0.16}}^{\#}$	
Left ventricular					
Hind wall thickness (µm)	1785 ± 44	1509 ± ${\mathrm{79}}^{\#\,\#}$	1924 ± 44	1581 ± ${\mathrm{72}}^{\#\,\#\,\#}$	
Septum thickness (µm)	1362 ± 34	1224 ± ${\mathrm{68}}^{\#}$	1396 ± 29	1292 ± 69	
Right ventricular					
Wall thickness (µm)	906 ± 28	793 ± 35	937 ± 42	850 ± 51	n.s.

^#^,^##^, ^###^, diabetic different from non-diabetic, *p *< 0.05, *p *< 0.01, *p *< 0.001. ^†^, TNC KO different from Wt *p *< 0.05, *p *< 0.01, *p *< 0.001. The numbers of animals in Wt non-diabetic, Wt diabetic, TNC-KO non-diabetic and TNC-KO diabetic groups were 11, 9, 10, and 7, respectively.

### 3.2 General Shapes of the Networks and Morphological Abnormalities

**Fig. 1.**
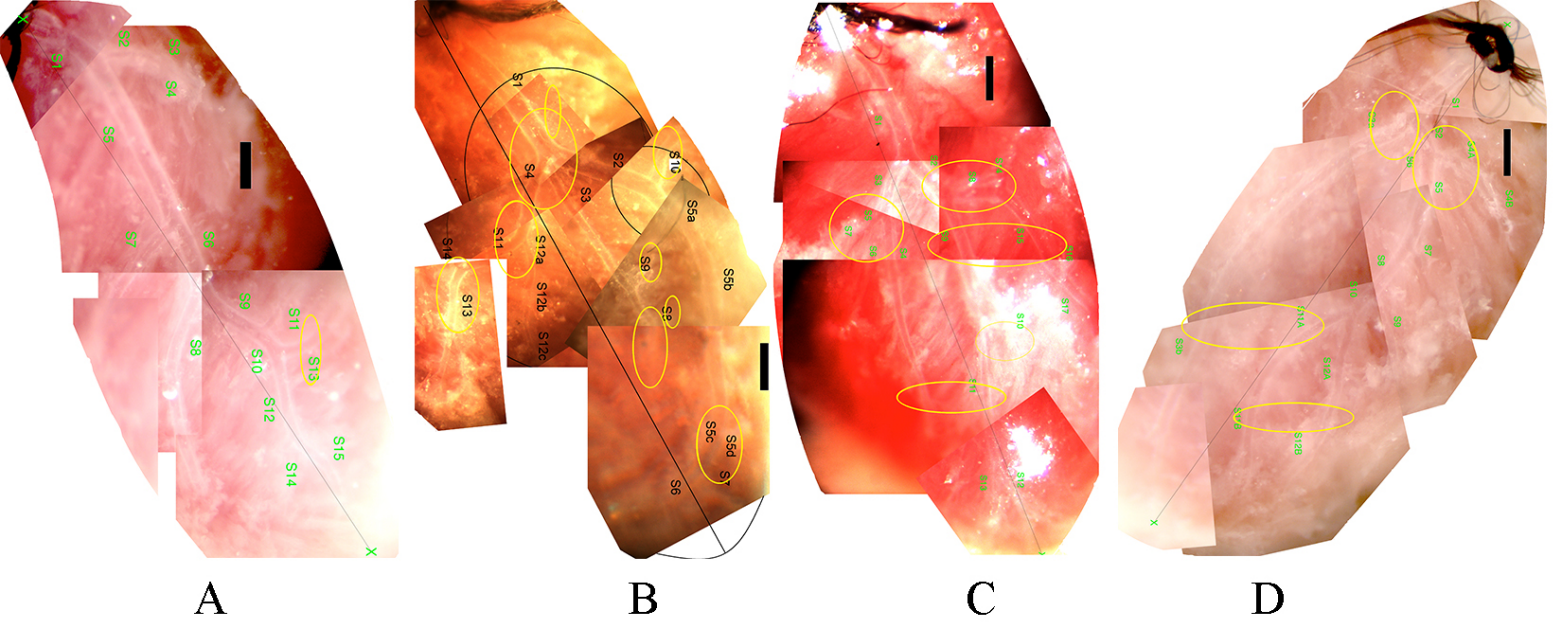
**Typical left coronary artery networks of mice, microprepared and *in situ* perfused**. (A) Wild type (A/J) non-diabetic. (B) Wild type (A/J) diabetic. (C) Tenascin C KO non-diabetic. (D) Tenascin C KO diabetic mice. Bars, 500 μm. Network abnormalities spotted are marked with ellipses (See text and Fig. 2).

**Fig. 2.**
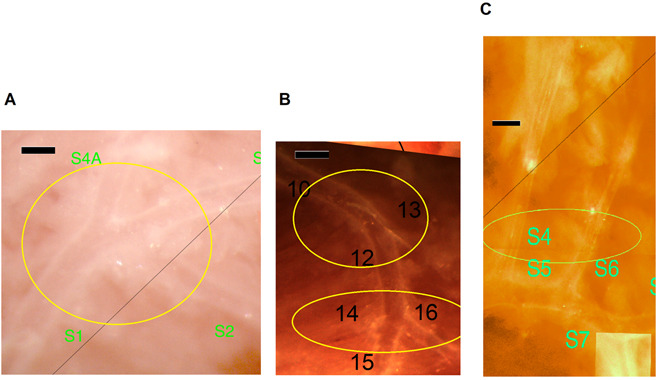
**Morphological abnormalities of the coronary arteriole network at higher magnification**. (A) Trifurcation (from a Wt diabetic mouse). (B) Trifurcation and bending of a larger branch (from a Wt diabetic mouse). (C) Larger branches running close to each other in parallel (from a TNC KO non-diabetic mouse). Bars, 200 μm.

Successful network preparations were made of 11 Wt non-diabetic, 9 Wt diabetic, 10 TNC KO nondiabetic and 7 TNC KO diabetic mice. Fig. 1 shows a typical network from each group. Morphological features often observed in the diabetic heart, include trifurcations, sharp bends of a larger branch, and branches leading in the retrograde direction with a vectorial component toward the orifice (Fig. 1B). In contrast, TNC KO mice with diabetes did not show significant elevation of the number of any such deformities significantly (Fig. 1D). The respective vascular “abnormalities” are shown at a higher magnification in Fig. 2 and the data summarized in Table 2.

**Table 2.** **Number of geometrical aberrations in coronary arteriole networks**.

	Wt		TNC KO	
	Non-diabetic (n = 11)	Diabetic (n = 9)	Non-diabetic (n = 10)	Diabetic (n = 7)
Trifurcations	2	${\mathrm{14}}^{\#\,\#\,\#}$	$8^{†}$	11
Sharp bending	7	${\mathrm{25}}^{\#\,\#\,\#}$	11	13
Retrograde branch	0	$9^{\#\,\#\,\#}$	0	3
Parallel branches	1	2	$9^{†}$	3

χ^2^ test ^###^, diabetic different from non-diabetic, *p *< 0.001. ^†^, TNC KO different from Wt *p *< 0.05, *p *< 0.01, *p *< 0.001.

### 3.3 Bifurcations

**Fig. 3.**
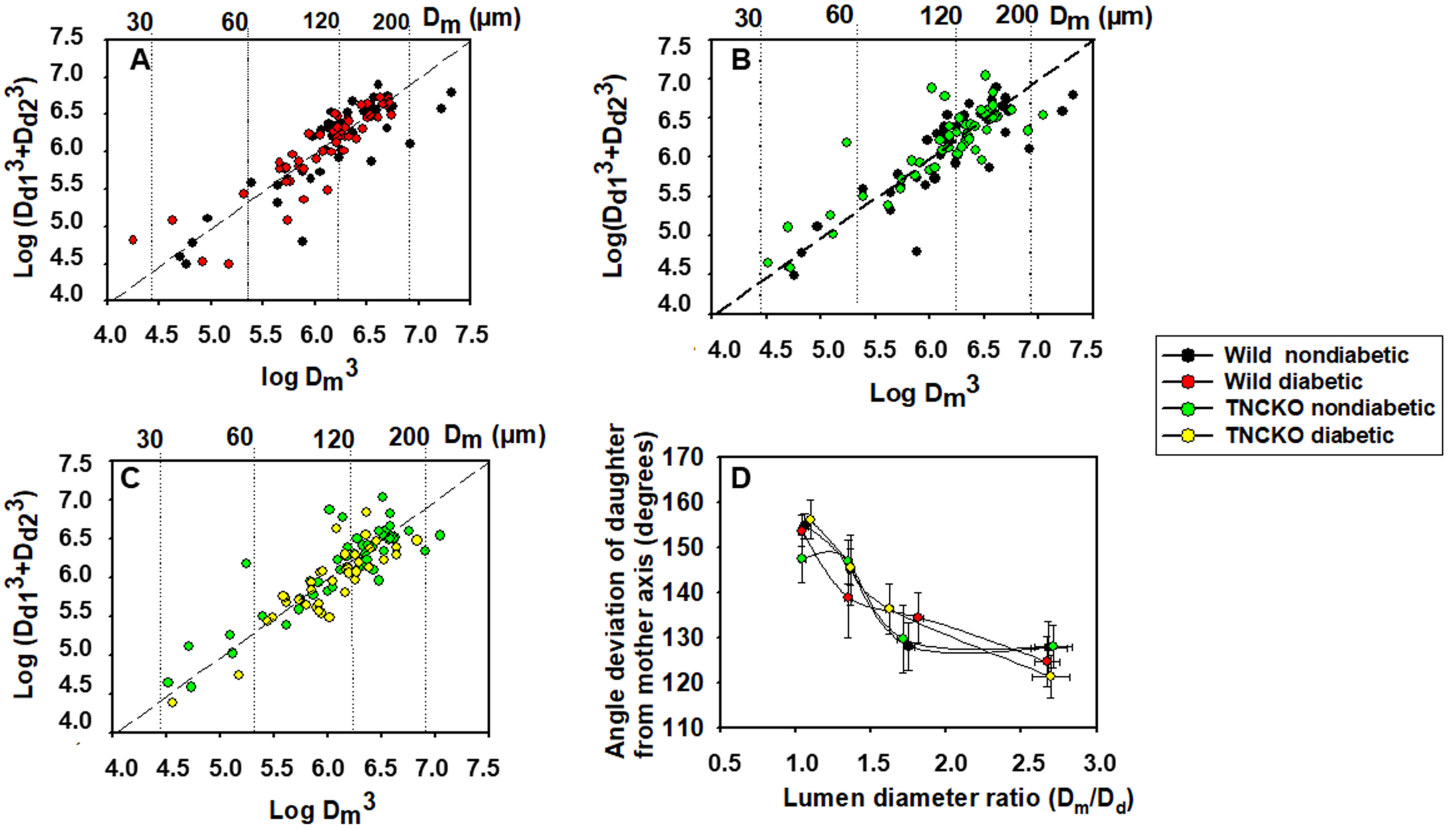
**Geometric analysis of bifurcations in coronary arteriole networks**. Sum of the cube of daughter branch diameter plotted against the cube of the diameter of the mother branch. Logarithmic scales. The Murray–law states that cube of the lumen diameter of the mother branch ($D_{\text{m}}$^3^) should be equal with the sum of the cubes of lumen diameters of the daughter branches ($D_{\text{d1}}$^3^+$D_{\text{d2}}$^3^). Scattered line corresponds to the validity of the law. (A) Adherence to the Murray-law compared for Wt non-diabetic and diabetic groups. (B) Adherence to the Murray-law for nondiabetic Wt and TNC KO mice. (C) Adherence to the Murray-law for nondiabetic and diabetic TNC KO mice. Scatters from the x = y line were not different with the F probe in any of pairwise comparisons. (D) Angle of the axis of the daughter branch with that of the mother branch as a function of the ratio of lumen diameters ($D_{\text{m}}$/$D_{\text{d}}$). Note that in all the four groups, branches with smaller diameter tend to deviate more from the direction of the mother branch.

In all four groups, geometry of 187 bifurcations was analyzed. All four groups adhered to the Murray-law. This law states that the cube of the lumen diameter of the mother branch is equal with the sum of the cubes of lumen diameters of the daughter branches. Pairwise comparisons for nondiabetic and diabetic groups as well as for wild and TNC KO non-diabetic groups are shown in Fig. 3A–C. Scattered line shows the sum of points where this law is valid. Scatter from this line was not different for the four observed groups when compared with the F probe. Fig. 3D shows an analysis of angles. The angle of the axis of the daughter branch with the axis of the mother branch is plotted against the ratio of lumen diameters. Fig. 3 also shows that while larger daughter branches ($D_{\text{m}}$/$D_{\text{d}}$~1.00) tend to follow the course of the mother branch (~${\mathrm{180}}^{o}$), smaller branches ($D_{\text{m}}$/$D_{\text{d}}$~3–4) tend to deviate more, approximating the perpendicular (~90°) direction. However, there was no statistical difference between the strains (Wt vs TNC KO) and treatment conditions (non-diabetic vs diabetic).

### 3.4 Overall Number of Network Components

A total of 531 resistance artery vascular segments were identified in the microsurgical preparation subsurface left coronary artery network. Using the high magnification synthetized pictures (collages, Fig. 1) each segment was divided in 50 μm length ring units. Overall, 9646 ring units were identified and their outer, inner diameter, wall thickness, position in the orifice-apex coordinate system and the flow distance from the orifice were determined. The right-bottom diagram of Fig. 6B shows the outer diameter histogram of all ring units of the diabetic and non-diabetic, Wt and TNC KO mice. The diagram shows the diameter frequencies—the number of ring units in a certain outer diameter range—of pooled data. Both diabetes and TNC deletion substantially elevated the number of vascular components constituting the network. It is outstanding, that diabetic networks were composed of a much greater number of ring units in the 100–180 μm range in Wt mice. Lack of *TNC* gene induced a similar alteration in network development. TNC KO mice with diabetes showed a further elevation in the number of ring units in the 100–140 μm, while it was significantly reduced in the 140–220 μm ranges (*p *< 0.001 with the χ^2^ test).

### 3.5 Wall Thicknesses

**Fig. 4.**
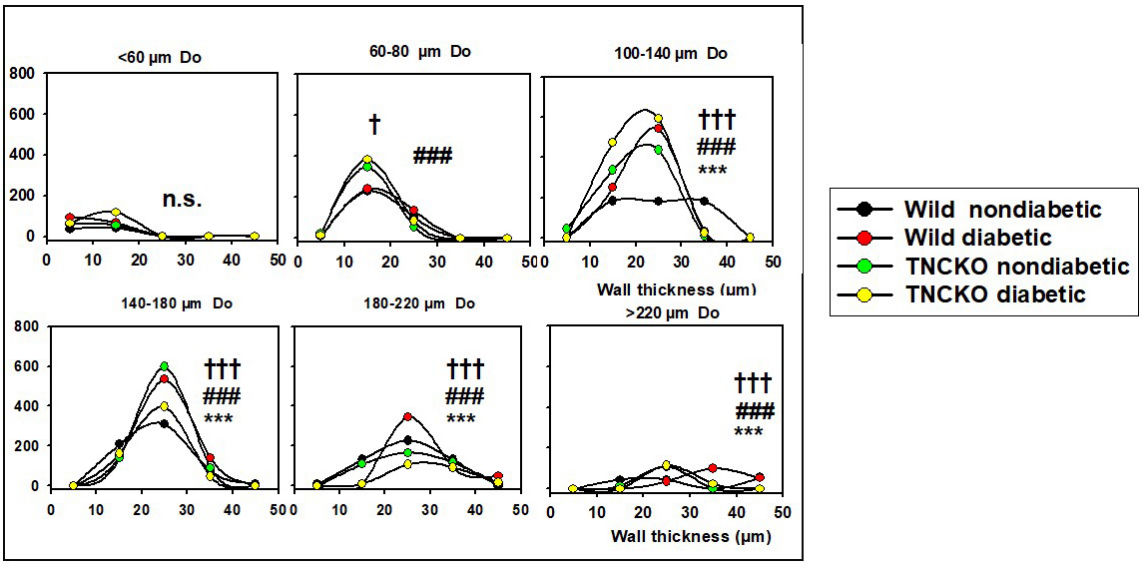
**Analysis of wall thicknesses**. All network was theoretically divided into 50 μm cylindrical ring units characterized (among others) by their diameter and wall thickness. Data extracted from 9646 ring units of 37 animals. Pooled data was normalized for 10 animals. Wall thickness histograms. Number of ring units (50 μm length) building up the networks in different outer diameter ranges with different wall thicknesses. Each plot represents a different outer diameter (Do) range. Wall thickness (h) ranges are plotted on the horizontal axis, pooled ring numbers on the vertical axis for each animal group. Differences were determined via χ^2^ test. ***, *p *< 0.001 between diabetic and non-diabetic Wt strain mice. ###, *p *< 0.001 between nondiabetic TNC KO and A/J strain mice. †, *p *< 0.05, †††, *p *< 0.001 between diabetic and nondiabetic TNC KO strain mice. Note that ring number differences appear in certain diameter and wall thickness ranges.

**Fig. 5.**
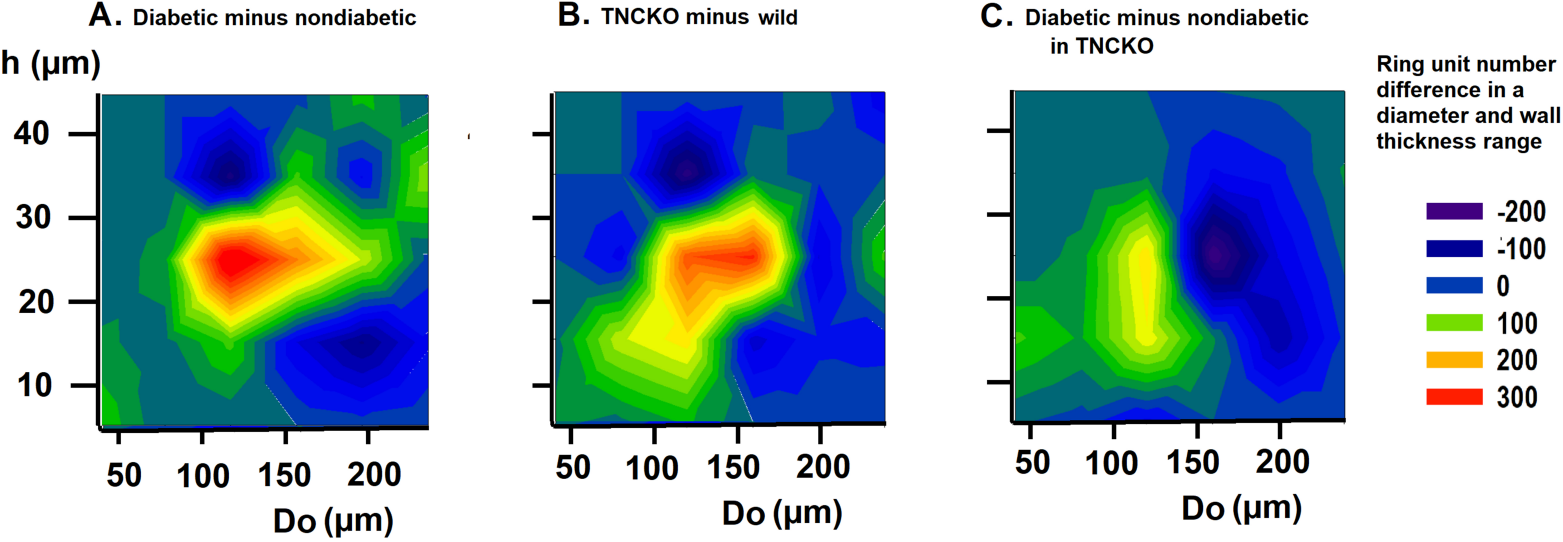
**Analysis of wall thicknesses**. Differences of bidirectional histograms, showing the difference in the number of ring units for different outer diameter (Do) and wall thickness (h) ranges between diabetic and non-diabetic Wt mice (A), TNC-KO and Wt non-diabetic mice (B) and diabetic and non-diabetic TNC KO mice (C). Further explanation see in Legend of Fig. 4. Red spots mark more, deep blue spots less ring units compared. Note that ring number differences appear in certain diameter and wall thickness ranges.

There were substantial changes in wall thicknesses upon diabetes and *TNC* deletion. Fig. 4 shows the wall thickness frequencies for different outer diameter ranges in the four groups. The thickening of the wall of largest vessels (>220 μm) from 20–30 to 30–40 μm in diabetes in Wt mice is one of the most important observations (hypertrophic segmental remodeling). There is a substantial elevation in the number of ring units in the 100–180 μm range with relative thin walls of 20–30 μm (vasculogenesis). The 100–140 μm units with thicker walls (30–40 μm) practically disappear marking a wall thinning process in this range (hypotrophic segmental remodeling). The diagram of Fig. 5A which demonstrates the difference between diabetic and non-diabetic (Wt) mice 2D histograms for diameter and wall thickness, reveals that there is a substantial elevation in numbers of ring units with a 100–180 μm outer diameter and a 20–30 μm of wall thickness (vasculogenesis). TNC KO caused a similar rearrangement (Fig. 4 green symbols, Fig. 5B), with even more intensive formation of new vascular units. However, diabetes failed to induce dramatic changes in TNC KO mice (Fig. 4, yellow lines, Fig. 5C). These alterations can be explained by the shrinkage of 140–180 μm vessels with maintained wall thicknesses.

### 3.6 Flow Distances

**Fig. 6.**
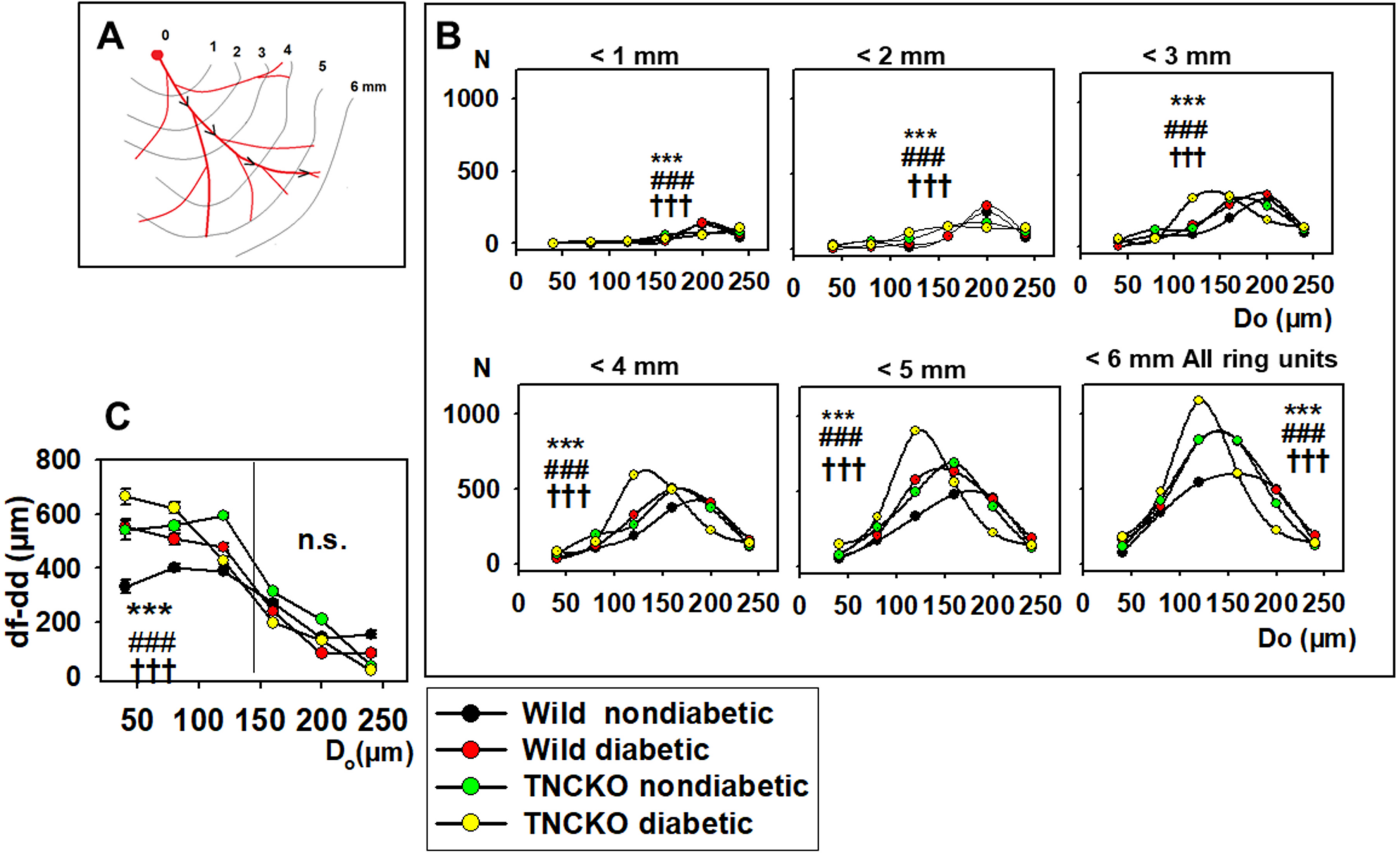
**Analysis of flow distances**. (A) Flow distance is defined as a distance that blood should flow from the orifice to the given ring unit. Data was extracted from 9646 ring units of 37 animals, pooled, and normalized for 10 animals in each group. (B) Diameter distribution (histogram) of ring units with flow distances (df) 1….6 mm from the orifice. Curves <6 mm demonstrate the full number of ring units in the given group. Note that ring number differences between animal groups appear in certain diameter and flow distance ranges. Significances for the χ^2^ test are shown. ***, *p *< 0.001 between diabetic and non-diabetic Wt mice. ###, *p *< 0.001 between non-diabetic TNC KO and Wt mice. †††, *p *< 0.001 between diabetic and non-diabetic TNC KO strain mice. (C) Flow distance minus direct distance values of ring units in the different diameter ranges for the four groups. Significances for the two-way anova test are shown. ***, *p *< 0.001 between diabetic and non-diabetic Wt mice. ###,* p *< 0.001 between non-diabetic TNC KO and Wt mice. †††, *p *< 0.001 between diabetic and non-diabetic TNC KO strain mice. Note that it is the wild-type nondiabetic group where blood flow must cover the minimum additional distance to reach the given vascular ring unit.

**Fig. 7.**
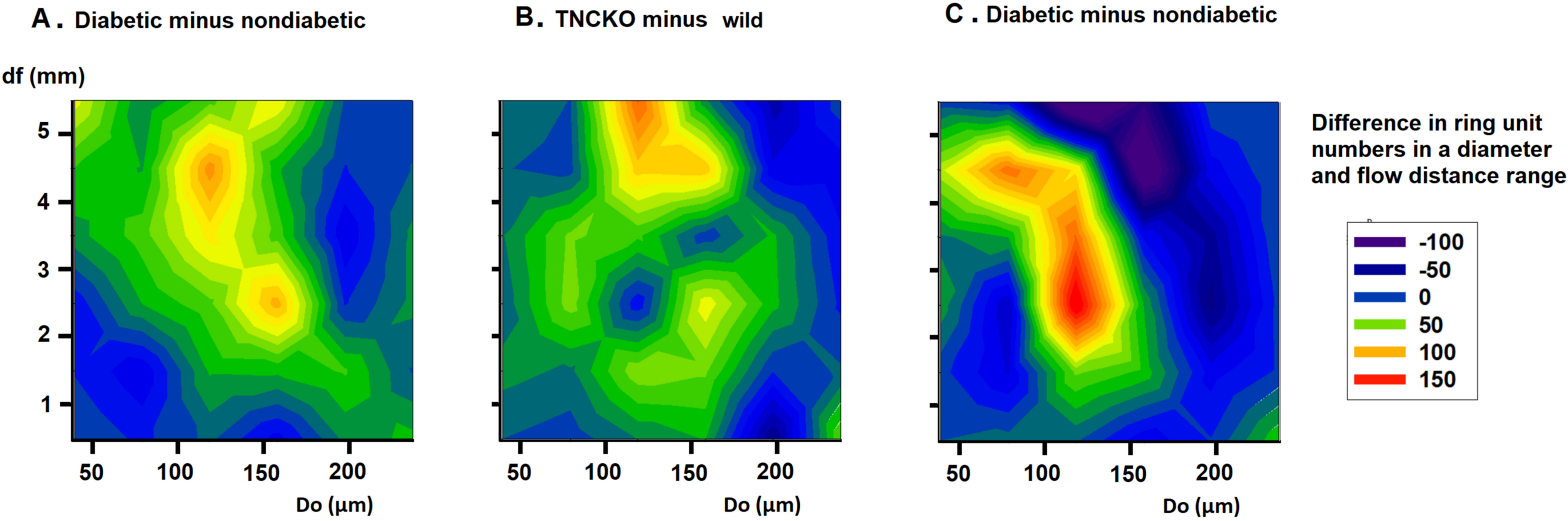
**Analysis of flow distances**. Differences of bidirectional histograms, showing the difference of the number of ring units for different flow distance ranges between diabetic and non-diabetic Wt mice (A), TNC-KO and Wt non-diabetic mice (B) and diabetic and non-diabetic TNC KO mice (C). Further explanation see in Legend of Fig. 6. Red spots mark more, deep blue spots less ring units compared. Note that ring number differences appear in certain diameter and flow distance ranges.

We investigated in what regions of the network such alterations did take place in the diabetic and TNCKO heart. Thus, outer diameter frequency histograms have been constructed for different flow distance ranges. Fig. 6B shows the frequency of vascular components in distances less than 1… 6 mm flow distance from the orifice for the non-diabetic Wt, diabetic, Wt non-diabetic, TNC-KO non-diabetic and diabetic TNC KO groups. Fig. 7 provides the difference of the number of ring units in a certain flow distance and diameter range between two groups (differences of two-dimensional histograms). Prominent is the elevation in the number of 100–200 μm elements at 2–5 mm flow distance from the orifice in diabetic wild types (Fig. 6 reed and black signals, Fig. 7A red spots) (*p *< 0.001 with the χ^2^ test). The elevated number of 100–180 μm units far from the orifice seems to be the most important consequence of the TNC deletion (Fig. 6 green and black signals, Fig. 7B, red spots). Increasing number of 100–140 μm elements at a 2–3 mm distance from the orifice, seeming to originate from shrinkage of originally 140–180 μm units, is caused by diabetes in TNC KO mice (Fig. 6 yellow and green symbols, Fig. 7C, red spots).

## 4. Discussion

Our results present new evidence for substantial alterations in the network geometry of the left coronary artery tree in a mouse model of STZ-induced diabetes. Here we also show that lack of extracellular matrix protein TNC effects coronary network formation and prevents malformations of network geometry and vessel wall thickness. Our data demonstrate for the first time that diabetes in mice results in (1) morphological deformations in the network associated with trifurcations instead of bifurcations, sharp bends of larger branches, and retrograde branches, (2) thickening of the wall of the largest diameter branches (>220 μm), that is the main left anterior descending coronary artery close to the orifice, thin walled larger branches practically disappeared, and (3) the number of medium-sized components (100–180 μm) substantially increased, they had characteristic wall thicknesses (20–30 μm) for that range of vessels and were located at their characteristic region (2–5 mm from the orifice). Finally, the wall thickness was reduced in the 100–140 μm range. Interestingly, diabetes did not affect the ability of bifurcations to form daughter branches of proper lumen and angle. Thus, a combined network remodeling was observed comprising with hypertrophic remodeling, hypotrophic remodeling and vasculogenesis at different segments of the network.

Literature search on on diabetic coronary resistance artery networks did not result in a comparable study. Of note, similar morphological deformations in the coronary resistance artery system, namely sharp bends of larger branches, trifurcations and parallel-running larger branches have been found previously in aged [17] and hypertensive [18] rats. Importantly, a common phenomenon of aging, hypertension, and diabetes is increased neuro-humoral activity including the renin-angiotensin-aldosterone system (RAAS). Activation of RAAS, particularly ACE 1 is a known regulator of vascular remodeling. Interestingly, a recent study highlights the interaction between ACE and TNC [28], which promotes adverse left ventricular remodeling.

Thickening of the coronary arteriolar wall is considered a characteristic pathological diabetic change [13, 14, 39, 40]. Segmental observations of diabetic coronary microvessels revealed inward hypertrophic remodeling in *db*/*db* diabetic mice which could be dependent on the angiotensin type 1 receptor [9] which is in concordance with our observations in the larger (>220 μm) diameter range of vessels. Previously, no wall thickness alterations were found in coronary arterioles of the 100s μm diameter range from diabetic patients, unless they show hypertensive responsiveness [15]. This is consistent with our findings on coronary arteries in the range of 100–180 μm in diabetic mice. Vascular neoformations in the forms of microaneurysms and spiral deformations in human diabetic specimens were described [12]. These findings are analogs with our observations, including trifurcations and sharp bends of larger branches. Diabetic neovascularization is typical in the retina and its clinically relevant pathological tortuosity is well-known [41]. Ophthalmoscopic photography is an excellent method to study geometrical characteristics of diabetic retinal arteriolar networks, while development of statistical methods for reproducible analysis is underway [41, 42]. We assume that a similar approach can be applied to coronary resistance artery network geometry. Because our approach relied on the STZ-induced diabetic mouse model, recapitulating the symptoms of type 1 diabetes. Therefore, further studies are warranted to clarify whether these geometrical alterations and remodeling of coronary resistance artery are identical in the type 2 diabetes.

TNC deletion was associated with early division followed by long, parallel running medium-sized branches, the number 100–140 μm branches was substantially elevated. Bifurcations were of proper diameter and angle. In addition, moderate thinning of the wall of 100–140 μm vessels was likely. Recent studies have demonstrated that higher TNC expression in cardiac tissue and its presence in plasma are associated with worse outcomes in patients with diabetes, however the role of TNC is still unknown [33, 43]. Our previous studies clearly demonstrated that the upregulation of TNC was associated with the progression of heart failure following myocardial infarction, chronic pressure overload and Duchenne Muscular Dystrophy [28, 29, 44]. Importantly, TNC KO diabetic mice did not induce wall thickness alterations, suggesting the pivotal role of TNC in diabetic maladaptive coronary artery remodeling which is more attenuated during the progression of diabetic cardiomyopathy. Additional investigations should reveal whether TNC KO mice are similarly protected against cardiac microvascular endothelial dysfunction in diabetes.

Vascular complications, particularly microvascular dysfunction are well-defined, substantial contributors to cardiac dysfunction in diabetes. Numerous studies indicate that metabolic dysfunction due to hyperglycemia promotes cardiac microvascular endothelial dysfunction [45]. Besides the effect of diabetes on cardiac microvascular endothelial dysfunction, the changes of microvascular geometry and network of the small resistance coronary arteries are similar to those observed in retinal vasculature [46]. Alternation of resistance artery geometry and increase of wall thickness certainly contribute to worsened cardiac perfusion and substantially to the progression of diabetic cardiomyopathy.

Considering the role of TNC in the formation of the coronary arteriolar network, we observed that this protein shapes the geometry of the coronary resistance artery network. Early division of the main branch, larger branches running parallel and close to each other were characteristic features in TNC KO animals. TNC is an embryonic protein, however its expression is resumed in cancerous tissue [47, 48], in chronic inflammatory processes [24] and even in proliferative diabetic retinopathy [49]. It interacts with several matrix proteins and inhibits cell adhesion through fibronectin, while also stimulates the expression of this cell adhesion protein [50]. TNC also contributes to fibrosis [24], and aggravates fibrotic remodeling in cardiac tissue after myocardial infarction [51]. Fibronectin-TNC aggregates are normally not present in the basement membranes but upon formation cause basement membrane thickening of retinal vessels in diabetic retinopathy [7]. Our present study revealed that lack of TNC KO resulted in a geometrically altered coronary resistance artery network that was characterized by early branching of the left anterior descending coronary artery. The emerging branches ran parallelly, in close proximity with each other, which resembles a “parallel fragmentation” of the network. Most surprisingly, STZ-induced diabetes did not induce further geometrical changes in TNC KO mice, suggesting that the re-expression of TNC in diabetes may constitute a key signaling molecule in the development of microvascular dysfunction in small coronary arteries.

## 5. Conclusions

Our data provide the first insight into the diabetic microvascular damage of coronary arterioles in a mouse model of STZ-induced diabetes. This revealed substantial changes in network geometry of coronary resistance arteries in diabetes and in mice lacking TNC expression. In diabetes, wall thickness of the largest branches increased (hypertrophic wall remodeling), the number of medium-sized vessels substantially increased (vascular neoformation), while wall thickness of smaller vessels decreased (dystrophic wall remodeling). In the present study, *in situ* perfusion, video-microscopic technique combined with the analysis of ring unit frequencies made it possible to demonstrate that these geometrical alterations appear in the characteristic diameter ranges of small arteries and arterioles; larger vessels closer to the orifice are affected in a different manner than smaller ones farther from it. This segmental specificity of diabetic microvascular pathology might be the most interesting observation. Surprisingly, the geometry of bifurcations showed no alterations, however large number of morphological malformations, trifurcations, sharp bends, and retrograde branches were found. Radial fragmentation of the network seems to be the main component of the pathology. We are convinced that such profound changes in network geometry contribute to the development of ventricular failure, they elevate the energy requirement of tissue oxygenation and disturb the adjustment of local flow patterns. Furthermore, TNC has an important role in forming coronary arteriole network geometry and certainly plays a causative role in the vascular wall thickening and remodeling, substantially contributes to microvascular dysfunction in diabetes.

## Data Availability

The data that support the findings of the present study are available from the corresponding author upon request.
